# Pancreatic Metastases of Rectal Cancer—Case Report and Literature Review

**DOI:** 10.1007/s12029-017-0016-9

**Published:** 2017-10-26

**Authors:** Tomasz Olesinski, Joanna Milewska, Malgorzata Symonides, Jakub Palucki, Andrzej Mróz, Andrzej Rutkowski

**Affiliations:** 1 Department of Gastroenterological Oncology, Maria Sklodowska-Curie Institute Cancer Center, Warsaw, Poland; 2 Department of Anesthesiology, Central Clinical Hospital of the MSWiA, 137 Wołoska Str, 02-507 Warsaw, Poland; 3 Department of Anesthesiology, Maria Sklodowska-Curie Institute Cancer Center, Warsaw, Poland; 4 Department of Radiology, Maria Sklodowska-Curie Institute Cancer Center, Warsaw, Poland; 5 Department of Pathology, Maria Sklodowska-Curie Institute Cancer Center, Warsaw, Poland

## Introduction

Colorectal cancer (CRC) is a significantly increasing cause of cancer mortality, being now the second most common malignancy in both genders. According to some sources, morbidity associated with summed cancers of the colon and rectum in the European Union population is even higher than morbidity associated with lung cancer or breast cancer [1]. Every year, more than 500,000 CRC deaths are noted worldwide. Thirty-five percent of newly diagnosed patients are in stage IV, i.e. a late stage of the disease with distant metastases [2]. Common metastatic sites of CRC include regional lymph nodes, the liver, the lungs and bones, whereas pancreatic metastases are rare. Secondary tumours account for only about 2% of all pancreatic malignancies [3]. A vast majority of patients with metastatic tumours within the pancreas also present with other distant metastases, and in this group, the only line of treatment is systemic palliative care. Nevertheless, there is a very small group of patients with an isolated metastatic tumour within the pancreas who may be referred for pancreatectomy, which may increase survival and positively affect prognosis. [3, 4].

## Case Report

We present the case of a 69-year-old woman who in May 2005 was diagnosed with adenocarcinoma of the rectum located 6 cm proximally from the anal verge. Concomitant diseases included type II diabetes and a long history of cigarette smoking. Using imaging diagnostics, the tumour was assessed as cT3N0M0. Initially, marker values were slightly elevated: CEA 7.1 ng/ml (normal range 0–3). The patient underwent preoperative irradiation of the pelvis using a three-field technique (X15 MeV photons; 5 fractions of 5 Gy/t up to a total of 25 Gy/t) followed by anterior resection of the rectum. Histopathology revealed very good response to radiotherapy with only few cancer glands invading maximally to muscularis propria (minimal residual disease) and displayed no nodal metastatic involvement. Postoperative period was complicated by anastomotic leakage—anastomosis was excised and sigmoidostomy was performed. After treatment, CEA concentration decreased to normal values. The patient entered oncological follow-up with routine screening for markers and computed tomography (CT) scans. In January 2008, CEA increased to 6.5 ng/ml, and reaching 17 ng/ml in September 2008.

**Fig. 1:**
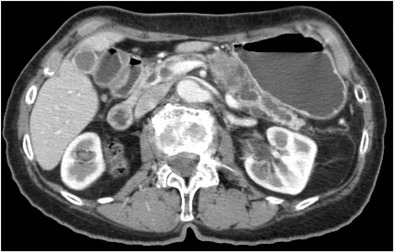
CT scan on the pancreatic lesion

In order to localise the site of recurrence, we performed abdominal and pelvic ultrasound (no pathological findings), bone scintigraphy (no pathological findings) and PET-CT, which presented an area of abnormal radiotracer density within the pancreas and (less suspicious) within the retroperitoneal space. We observed further increase of CEA to 18.7 ng/ml (September 2008) and a normal level of CA-19.9. Due to dissemination, patient was referred to chemotherapy according to the LF3 protocol (LV 36 mg, 5FU 756 mg administered for 2 days every 2 weeks). After the 7th course of chemotherapy, CEA concentration increased to 27.9 ng/ml and CA-19.9 level remained 0 IU/ml. We performed a control CT scan in which an irregular mass of approx. 30 × 30 mm was found between the corpus and tail of the pancreas (Fig. 1). Other tissues and organs presented no abnormalities.

**Fig. 2:**
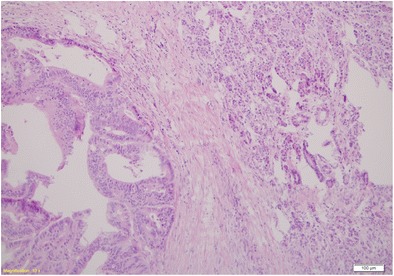
Pathological presentation of the pancreatic metastasis (left side) and pancreatic tissue (right side): staining H&E, (× 100)

**Fig. 3:**
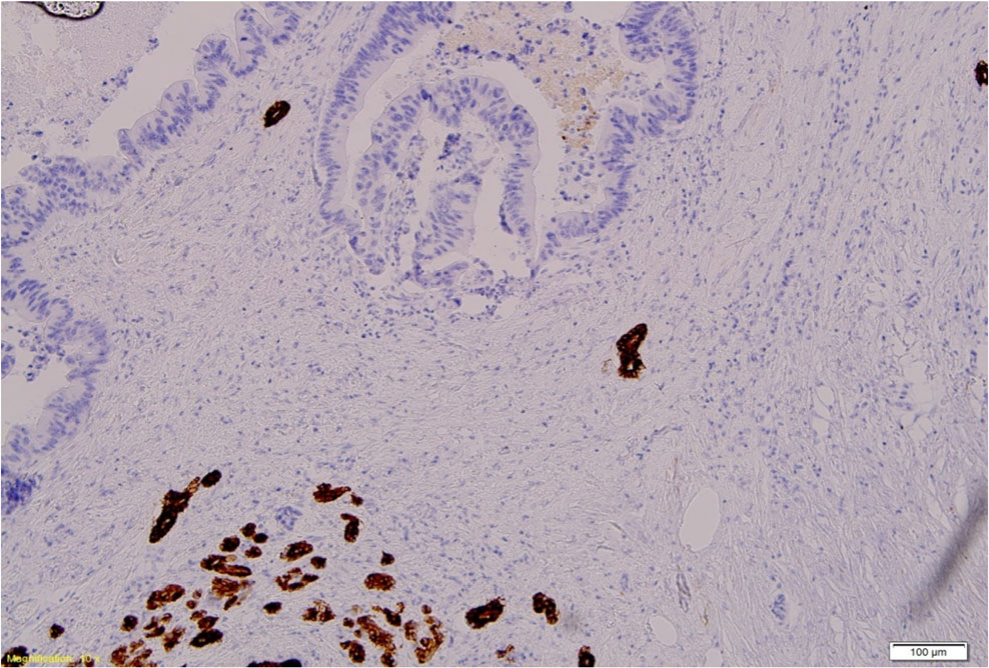
Pathological presentation of the pancreatic metastasis; staining CK7, (× 100)

**Fig. 4:**
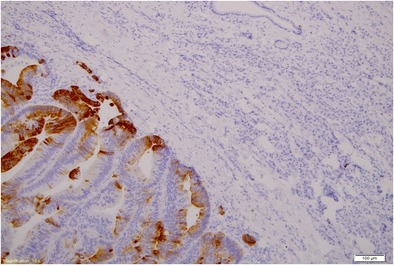
Pathological presentation of the pancreatic metastasis; staining CK20 (× 100)

**Fig. 5:**
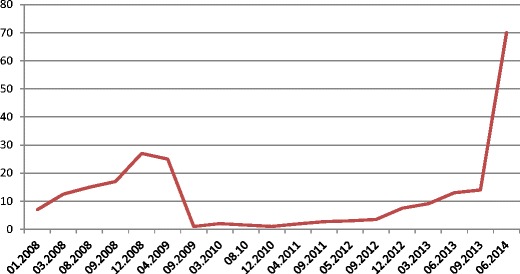
Changes in CEA level in the course of follow-up

The patient was referred for surgery. In September 2009, she underwent distal resection of the pancreas with concomitant splenectomy. During surgery, no symptoms of intraperitoneal dissemination were found and the parapancreatic lymph nodes were unchanged. Recovery was uneventful. On macroscopic and microscopic examinations, a relatively well-delineated noncapsulated tumour was found. It consisted of atypical glands of relatively large size lined with high cylindrical epithelium. Glands’ lumina were filled with necrotic material reminding so called dirty necrosis as in colon cancer. Also, epithelium looked similar to intestinal lesion even though the colon cancer was changed by preoperative treatment. In order to confirm intestinal origin, immunohistochemistry was performed to reveal CK20 positivity and CK7 negativity as in colon cancer immunoprofile. In addition neighbouring parenchyma of the pancreas was unremarkable with reverse profile (CK7+, CK20-). The diagnosis of rectal adenocarcinoma metastases was made. The excision was complete (R0) (Figs. 2, 3, 4). After surgery CEA fell from 30 ng/ml to normal values. The patient received adjuvant treatment according to XELOX protocol (oxiplatin 226 mg, xeloda 2 × 1500 mg per die over 14 days). Due to symptoms of neurotoxicity (dizziness, nausea, vomiting) only one course of chemotherapy was administered. According to the patient’s request the LF3 protocol was recommenced (LV 34 mg, 5FU 634 mg over 2 days). Altogether, between January and June 2010 she had been administered with 12 courses of chemotherapy. During treatment marker levels were low (fig. 5). From the termination of adjuvant treatment until the end of February 2013 the patient was in routine follow-up. On the 20th of February 2013 PET-CT was performed due to an elevation of markers with no other symptoms of disease. It showed metabolically active infiltrates in the pre-sacral area, within the uterine wall and in the 11 thoracic vertebra and a tumour in the right lung. Bronchoscopy revealed an exofitic comedo infiltration closing the lumen of the 8th segment (pathology: rectal cancer metastasis). The patient received palliative treatment which ended in October 2014 due to progression (metastases to the CNS, thyroid and left suprarenal gland) with referral for symptom relief.

## Discussion

The risk of colorectal cancer increases over the age of 50 and reaches its peak in subjects over 70 years of age [2]. Colorectal cancer develops slowly. It may take over 10 years for small dysplastic lesions within the colorectal epithelium to grow into cancer. Nevertheless, in some 35% of patients, the disease is diagnosed at disseminated stage, while in almost 50% of cases in clinical stage II and III, dissemination will take place during the follow-up period [5]. The tumour is characterised by lymphatic spread to the lymph nodes, continuous spread through the colorectal wall and along the mucous membranes and along blood vessels [6]. Metastases are usually found within the local lymph nodes (50–70%), the liver (50%), the bones (40%), the lungs (21%), the peritoneum (15%), and, less often, within the ovaries (15%) and the brain (5%) [2, 7]. Development of cancer metastases is a complex process dependent on the characteristics of the neoplastic cell, such as proteolytic and proliferative activity and ability to migrate and promote neovascularization.

Pancreatic metastases of colorectal cancer are particularly rare. Until now, only a few cases have been reported. Other malignancies which may metastasize to the pancreas include renal cancer, malignant melanoma, lung cancer, breast cancer, gastric cancer and cancer of the small intestine [8, 9]. Secondary malignancies account for some 2–3% malignant pancreatic lesions. The most common pancreatic malignancy is ductal adenocarcinoma (85%)—a primary malignant tumour of the pancreas [9]. The diagnosis of pancreatic lesions—especially in early stages—is highly problematic. In some 90% of cases, the tumour is already non-resectable at the time of diagnosis because clinical symptoms appear at a very late stage, while the localisation of the pancreas renders it difficult to analyse, especially by ultrasound. Endosonography is a tool of relatively high sensitivity and specificity for pancreatic imaging—it allows both tumour visualisation and biopsy, however its availability is somewhat limited [10, 16]. Generally, the diagnosis of pancreatic tumours and their dissemination is based on ultrasound, computed tomography and magnetic resonance. PET-CT may also be a helpful modality [11].

Early pancreatic metastases, just as primary pancreatic tumours, should be treated surgically. However, a vast majority of diagnoses are made at a very advanced stage with infiltration of local organs, local nodal involvement or distant metastases. Only some 20% of patients may be referred to surgery [10]. The scope of surgery depends on tumour localisation—total pancreatectomy, pancreatoduodenectomy (Whipple procedure)—i.e. resection of the head of the pancreas with the duodenum and distal part of the stomachor distal pancreatectomy (partial distal resection of the pancreas). Palliative surgical procedures are performed in order to provide adequate biliary or alimentary passage. These include biliary-intestinal anastomoses which allow bile evacuation and resolve mechanical jaundice and gastrointestinal anastomoses which allow adequate alimentary flow through the infiltrated duodenum [12]. If the tumour is located in the proximity of larger local veins, e.g. the upper mesenteric vein, it is possible to dissect the infiltrated part of the vessel and to transplant a graft obtained from the lower extremities, e.g. the superficial femoral vein [13].

While in the case of primary pancreatic tumours, basic treatment is surgery; this approach is still being discussed in the case of secondary pancreatic malignancies. An analysis of literature provides only several hundred of cases discussing various secondary pancreatic tumours. The rationale of metastatic tumour resection is limited only to cases with no symptoms of dissemination beyond the pancreas [14]. A 5-year survival ratio after resection of an isolated colorectal metastasis to the pancreas exceeds 50% [4]. If, at this stage, colorectal cancer is already generalised, the treatment of choice is palliative chemotherapy or surgical anastomoses to preserve free passage along the gastrointestinal tract.

Pancreatic metastases of colorectal cancer are very rare and extremely difficult to diagnose, while delayed diagnosis affects prognosis negatively [10]. In the presented case, diagnosis was commenced with the increase CEA concentration. Abdominal and pelvic ultrasound and scintigraphy did not reveal any abnormalities. Only PET-CT imaging provided suggestions as to the presence of a metastatic tumour within the pancreas. On histological grounds, it was mandatory to differentiate primary pancreatic adenocarcinoma from colorectal cancer metastasis. Histological pattern of the glands, pronounced isolation of the tumour and uninvolvement of neighbouring pancreatic tissue were all in favour of metastasis diagnosis. Immunohistochemical profile confirmed colonic origin of cancerous lesion in the pancreas. It is difficult to assess the impact of pancreatectomy on treatment efficacy and further prognosis; however, some data from literature suggest that malignant tumour resection from the pancreas improves survival and, in some cases, may even provide full recovery [4, 15–17].

## Conclusions

In the case of an isolated metastasis of colorectal cancer to the pancreas, excision must be considered as a therapeutic modality.
